# Association of serum complement C3 with metabolic syndrome components in normal weight obese women

**DOI:** 10.1186/s40200-017-0330-6

**Published:** 2017-12-28

**Authors:** Maryam Karkhaneh, Mostafa Qorbani, Mohamad Reza Mohajeri-Tehrani, Saeed Hoseini

**Affiliations:** 1 Department of Community Nutrition, School of Nutritional Sciences and Dietetics, Tehran University of Medical Sciences, Tehran, Iran; 2 Non-communicable Diseases Research Center, Alborz University of Medical Sciences, Karaj, Iran; 3 Chronic Diseases Research Center, Endocrinology and Metabolism Population Sciences Institute, Tehran University of Medical Sciences, Tehran, Iran; 4 Endocrinology and Metabolism Research Center, Endocrinology and Metabolism Clinical Sciences Institute, Tehran University of Medical Sciences, Tehran, Iran; 5 Department of Clinical Nutrition, School of Nutritional Sciences and Dietetics, Tehran University of Medical Sciences, Hojatdost street, Naderi street, Keshavarz Blv., Tehran, Iran

**Keywords:** Normal weight obesity, Body fat mass, Complement C3, Metabolic syndrome

## Abstract

**Background:**

Increased serum complement C3 has been related to body fat mass, metabolic syndrome and chronic diseases. The purpose of this study was to evaluate the levels of C3 in the subjects of normal weight obese (hereafter NWO) as well as their possible relationships with metabolic syndrome and inflammation.

**Methods:**

In this case-control study, 40 obese women with normal weight (body mass index (BMI) = 18.5–24.9 kg/m^2^) and body fat percentage above 30% (fat mass (FM) > 30%) and 30 non-obese women (BMI = 18.5–24.9 kg/m2) and fat percentage less than 25% (FM < 25%) were selected as the study sample. Body composition was analyzed using Bio Impedance analyzer. Blood samples were then collected and analyzed for fasting serum concentration of lipid components of metabolic syndrome, insulin, serum complement C3 and High sensitivity C reactive protein (hsCRP).

**Results:**

Mean waist and hip circumferences in NWO was higher than non-NWO (74.78 ± 4.81 versus 70.76 ± 2.91 and 99.12 ± 4.32 versus 93.16 ± 2/91, respectively, *P*-value < 0.001). However, the mean waist-to-hip ratio did not differ significantly (*p* = 0.448). The mean fasting serum concentration of complement C3, hsCRP and insulin was higher in NWO compared to that in non-NWO (*P*-value < 0.05). Moreover, insulin sensitivity in NWO was lower than that in non-NWO (0.357 versus 0.374, *p*-value = 0.043). Moreover, a significant correlation was found between body fat percentage and fasting serum complement C3 and insulin concentration (*r* = 0.417 and *r* = 0.254, *p*-value < 0.005, respectively).

**Conclusion:**

Obese women with normal body mass index but high body fat percentage have higher serum C3 and are at a higher risk for metabolic dysregulation and metabolic syndrome than the healthy non-obese subjects.

## Background

Obesity is a public health problem in many countries [1–3]. Since it is an independent risk factor for metabolic syndrome, diabetes, and cardiovascular disease [4–6]. The prevalence of both obesity and metabolic syndrome is increasing on a global scale. The World Health Organization (WHO) defines obesity as an excessive accumulation of fat in the body to an extent that it affects the health [7]. The excess of body fat mass is a major source of cytokines, leading to a pro-inflammatory environment in the body [8]. Thus, for screening and diagnosis of obesity, the measurement of the body fat mass is more accurate than BMI [9].

A sub group of obese people called normal weight obese individuals has been identified in this regard [9]. Such individuals can be determined by a normal body mass index (18.5 ≤ BMI ≤ 24.9) and a high body fat percentage that shows the degree of metabolic dysregulation [10]. Accordingly, despite having a normal body mass index, they are potentially at an increased risk for the development of metabolic syndrome, cardiometabolic dysfunction, and higher mortality [11]. Previous studies have shown that body fat mass is associated with the high prevalence of metabolic syndrome and it̛s components in individuals with a normal body weight [12].

The complement system plays an important role in innate immunity mechanisms. The complement factor 3 (C3) is a key factor for the activation of the three complement activation pathways: classical, alternative, and mannose-binding lectin [13]. Indeed, complement C3 is an acute-phase reactant produced by the liver, activated macrophages in inflammation sites, and adipocytes [14]. The biological mechanism of C3 in adiposity is attributed to the C3- degradation product. C3 play role in adiposity biology from different mechanism including stimulates lipogenesis in adipocytes in interaction with insulin; stimulation of triglyceride synthesis and preventing the release of free fatty acids derived from lipolysis [15]. Findings from previous studies suggest a relationship between systemic C3 and adiposity. This link is verified by the observations that the adipose tissue secretes C3, that weight gain is associated with an increase in C3, and that C3 decreases upon weight loss [16]. Moreover, serum C3 levels are associated with intra-abdominal adipose tissue [17].

There is evidence supporting a potential role for circulating C3 in the development of obesity, type 2 diabetes, and cardiovascular diseases [18–21]. Moreover, C3 concentrations have been shown to be a useful biomarker to identify subjects with metabolic syndrome features [14, 22, 23] and that its increased concentrations, among other mediators such as CRP (C Reactive Protein) and ESR (Erythrocyte Sedimentation Rate), are strongly associated with insulin resistance [20], waist circumference, and both high fasting and postprandial triacylglycerol concentrations [17, 24].

This study sought to evaluate the serum C3 concentration in women with normal weight obese (NWO) syndrome compared to controls as well as the relationship between complement C3 and body fat mass and the components of metabolic syndrome. This line of research is very important because NWO individuals are frequently undetected and undiagnosed because of their normal BMI and younger age [25].

## Methods

### Subjects

This case-control study consisted of 70 Iranian normal weight women subdivided into two groups: 40 NOW individuals (BMI < 24.9 kg/m2 and fat mass (FM)% > 30) and 30 control age-matched women (BMI < 24.9 kg/m2 and FM% < 30).

The study was approved by the Medical Ethics Committee of Endocrinology and Metabolism Research Center of Shariati Hospital. The subjects were recruited by announcements in the Youth Sports Club. Among them, women aged 19–39 years with normal weight for height who had joined the club recently were selected to measure body composition using the BIA (BIO ELECTRIC IMPEDANCE ANALYSER).

The inclusion criteria for participation were female sex and age range of 19–39 years old with a normal body mass index. Furthermore, the exclusion criteria for participation were acute illness, diabetes, hypertension, dyslipidemia, liver diseases, kidney diseases, cardiovascular diseases, diseases associated with increased cortisol levels such as Cushing’s syndrome, thyroid disease, autoimmune diseases and infections, drug consumption, lactation, and pregnancy.

Almost all of the women were in the follicular phase of the menstrual cycle and did not smoke or abuse alcohol.

### Overview of the protocol

After reading and signing the written consent form, each participant was invited to receive a series of tests. The subjects arrived in a fasting state at 8:00 o’clock in the morning. Body composition analysis and anthropometric measurements were performed for each participant; then, a blood sample was taken for the determination of a fasting lipid profile and analyses of insulin, glucose, serum complement C3, and hsCRP.

### Anthropometric measurements

Height was measured without shoes using a digital meter mounted on a wall to the nearest 0.5 cm. Weight was measured by the TANITA body composition analyzer in the fasting condition with minimal clothes and without shoes, after defecation, at 8 to 9 am. The body mass index was calculated by dividing weight (kilograms) to height (meters squared), which was automatically performed by the TANITA analyzer. Waist circumferences (WC) and hip circumferences (HC) were measured with the use of a metric tape to the nearest 0.5 cm.

### Body composition

Analysis of body composition was carried out using the BIO IMPEDANCE method and TANITA Body Composition Analyzer (BC-418MA) in specific conditions such as fasting, not drinking too much water, as well as avoiding physical activity and exercise before the test, at 8 or 9 o’clock in the morning.

### Blood samples

Blood samples were collected and measured after overnight fasting (10–12 h) for serum concentrations of total cholesterol, high-density lipoprotein (HDL) cholesterol, low-density lipoprotein (LDL) cholesterol, triglycerides, glucose and insulin, complement C3 and hsCRP. Venous fresh blood samples (10 ml) were collected from the antecubital vein. 2 ml of the venous fresh blood samples was transferred to sterile tubes containing anticoagulant EDTA and the remaining 8 ml was poured into other vacuum tubes for faster coagulation. All materials were immediately placed in ice. Serum was obtained by centrifugation at 1500 rpm for 10 min. All samples were stored at −80 °C and were analyzed after 3–4 months. Serum total cholesterol, LDL cholesterol, HDL cholesterol, triglycerides, fasting glucose, complement C3, hs-CRP were measured by the 902 Hitachi autoanalyzer through the photometric method using Pars test kits. The serum Insulin concentration was determined using the ELISA method with the Monobind kit and HbA1C was measured using the D & 5 device and ion-exchange chromatography technique. Insulin resistance was determined applying homeostasis model assessment of insulin resistance, (HOMA-IR): fasting glucose (mmol/L) × fasting insulin(μIU/mL)/22.5 [8].Insulin sensitivity was measured by the index QUICKI using the following formula:

Quantitative Insulin Sensitivity Check Index = 1 / [log(fasting insulin μU/mL) + log(fasting glucose mg/dL)] [26].

### Statistical analyses

The data were analyzed running SPSS statistical software (version 16.0; SPSS Inc., Chicago, IL, USA). Normal distribution of continuous variables was checked by Kolmogrov-smirnov test. Continuous variables with normal distribution are reported as mean ± standard deviation (SD). Independent T-test was used to compare the mean of continuous variables that had a normal distribution. Median and interquartile range (IQR) was also used for continuous variables that were not distributed normally. The median of continuous variables that were not distributed normally was compared using Man-Withney U test. The Pearson correlation coefficient was used to assess correlation between continuous variables. In addition, univariate and multivariate logistic regression analysis was used to examine the relationship between the independent variables and NWO. In multivariate model all significant variables in the univariate model were included in the multivariate model. The results of logistic regression analysis are presented as odds ratio (OR) and 95% confidence interval (CI). *P*-value < 0.05 was considered as statistically significant.

## Results

Mean age (SD) in NOW and non- NOW was 28.45(4.87) and 28.83 (4.39) respectively which was not statistically significant. Table 1 shows the mean of anthropometric indices and blood pressure in NWO and non-NWO women. Weight, BMI, FM, waist and hip circumference (*p* < 0.001) differed significantly between the two groups.

**Table 1:** Mean and standard deviation of anthropometric indices and blood pressure in normal weight obese and non-normal weight obese women

	NWO (*n* = 40) Mean ± SD	Non-NWO (*n* = 30) Mean ± SD	*P* value
Age (y)	28.45 ± 4.87	28.83 ± 4.39	0.735
H (cm)	166.12 ± 4.45	165.68 ± 4.72	0.690
W (kg)	62.97 ± 4.92	57.14 ± 4.20	< 0.001
BMI (kg/m^2^)	22.67 ± 1.26	20.85 ± 1.32	< 0.001
FM (%)	32.75 ± 2.62	23.52 ± 1.68	< 0.001
FM (kg)	20.60 ± 2.92	13.41 ± 1.43	< 0.001
FFM (KG)	42.12 ± 2.83	43.52 ± 3.16	0.057
WC (cm)	74.79 ± 4.82	70.77 ± 2.91	< 0.001
HC(cm)	99.13 ± 4.32	93.17 ± 2.91	< 0.001
W/H	0.75 ± 0.04	0.76 ± 0.03	0.448
SBP (mmHg)	101.8 ± 0.67	99.2 ± 0.68	0.118
DBP (mmHg)	71.7 ± 0.78	69.1 ± 0.71	0.157

*H* Height, *W* Weight, *WC* Waist circumference, *BMI* body mass index, *FFM* fat free mass, *FM* fat mass, *HC* Hip circumference, *W/H* Waist to hip ratio, *SBP* Systolic blood pressure, *DBP* Diastolic blood pressure

### Biochemical parameters, insulin sensitivity and insulin resistance

Lipid parameters, fasting blood glucose, fasting serum insulin, insulin sensitivity, insulin resistance and inflammatory markers are given in Table 2. No significant differences were observed in total cholesterol, HDL cholesterol, LDL cholesterol, triglycerides and fasting blood glucose between the two groups. However, fasting serum insulin and hemoglobin A1C was statistically different between the two groups. Serum C3 and hs-CRP concentrations were higher in NWO than non-NWO. Furthermore, significant differences were found in insulin sensitivity (*p* = 0.003), and insulin resistance (*p* = 0.001) indices between the two groups.

**Table 2:** Metabolic parameters, insulin sensitivity and insulin resistance in normal weight obese and non-obese women

	NWO (*n* = 40)	Non-NWO (*n* = 30)	*P*-value
TC (mg/dl)^a^	174.75 ± 20.82	172 ± 93 ± 20.15	0.769
HDL_C_ (mg/dl)^a^	59.15 ± 13.38	60 ± 8.59	0.590
LDL_C_ (mg/dl)^a^	91.25 ± 17.69	87.3 ± 15.61	0.335
TG (mg)^a^	89.05 ± 29.16	81.5 ± 25.44	0.262
FBS(mg/dl)^a^	82.72 ± 8.02	85 ± 7.02	0.207
FSI (μIU/ml)^a^	9.03 ± 4.66	5.93 ± 2.11	< 0.001
HbA1C (%)^a^	4.687 ± 0.621	4.393 ± 0.402	0.027
C3 (g/l)^a^	105.62 ± 15.74	92.8 ± 8.48	< 0.001
hs-CRP* (mg/L)^a^	0.5 (1.275)	0.300 (0.425)	0.01
HOMA-IR^b^	1.88 ± 1.13	1.25 ± 0.48	0.001
QUICKI^a^	0.35 ± 0.27	0.37 ± 0.25	0.003

*TC* Total cholesterol, HDL_C_ HDL cholesterol, *LDL*_*C*_ LDL cholesterol, *TG* Triglycerides, *FBS* Fasting blood sugar, *FSI* Fasting serum insulin, *Hb A1C* Glycosylated hemoglobin, *C3* serum Complement C3, *hs-CRP* High sensitivity c reactive protein, *HOMA-IR* Homeostasis model assessment of insulin resistance, *QUICKI* Quantitative Insulin Sensitivity Check Index

^a^Data are reported as mean (standard deviation)

^b^Data are reported as median (interquartile range)

### The association between independent variables and normal weight obesity

Table 3 shows the crude and adjusted ORs of independent variables and normal weight obesity. In the univariate model, the association between all independent variables and NWO were statistically significant. In the multivariate model, all associations remained statistically significant except for weight, WC and hs-CRP (*p* > 0.05).

**Table 3:** Association between independent variables and normal weight obesity in logistic regression model

	Model 1 (crude)			Model 2 (adjusted)		
	OR	95% CI	*P*-value	OR	95% CI	*P*-value
W (kg)	1.352	1.16–1.58	< 0.001	1.157	0.96–1.4	0.135
C3 (g/l)	1.090	1.04–1.15	0.001	1.086	1.02–1.15	0.006
WC (cm)	1.296	1.11–1.51	0.001	1.103	0.92–1.32	0.290
HC (cm)	1.569	1.27–1.93	< 0.001	1.384	1.11–1.73	0.004
FSI (μIU/ml)	1.429	1.14–1.79	0.002	1.348	1.03–1.76	0.027
hs-CRP(mg/L)	2.636	1.11–6.28	0.029	2.227	0.8–6.18	0.124
HOMA-IR	1.082	1.02–1.15	0.007	1.071	1.0–1.15	0.046
Hb A1C	3.168	1.1–9.12	0.033	4.042	1.04–15.71	0.044

*OR* Odd ratio, *CI* confidence interval, *W* Weight, *C3* serum Complement C3, *WC* Waist circumference, *HC* Hip circumference, *SI* Serum insulin, *hs-CRP* High sensitivity c reactive protein, *HOMA-IR* Homeostasis model assessment of insulin resistance, *Hb A1C* Glycosylated hemoglobin

### Correlation between body fat percentages and complement C3 with anthropometric indices, blood pressure and metabolic parameters

Table 4 shows the correlation coefficient between the body fat percentages and complement C3 with independent variables. Both body fat percentages and complement C3 had significant correlation with fat free mass, fasting serum insulin, hs-CRP and insulin sensitivity (*p* > 0.05).

**Table 4:** Correlation coefficient between fat mass and complement C3 with anthropometric indices, blood pressure and metabolic parameters

	Variable																		
Correlation coefficient	WC	HC	W/H	SP	DP	C3	TC	LDLc	HDLc	TG	Weight	FFM	%FM	Hs CRP	QUI CKI	HOMA IR	Hb A1C	FSI	FBS
Fat mass %	0.221	**0.568	−0.225	0.072	−0.019	*0.417	0.079	0.101	0.069	0.097	*0.268	**−0.589	1	*0.275	*-0.260	0.195	0.142	0.254*	0.069-
C3	0.225	0.172	0.052	0.042	0.024	1	*0.315	*0.310	0.041	**0.473	0.039	*0.323	**0.417	*0.405	*-0.265	*0.305	0.050	*0.316	0.131

*WC* Waist circumference, *HC* Hip circumference, *W/H* Waist to hip ratio, *SP* Systolic blood pressure, *DP* Diastolic blood pressure, *C3* serum Complement C3, *TC* Total cholesterol, *LDL*_*C*_ LDL cholesterol, *HDL*_*C*_ HDL cholesterol, *TG* Triglycerides, *FFM* fat free mass, *%FM* fat mass percentage, *hs-CRP* High sensitivity c reactive protein, *QUICKI* Quantitative Insulin Sensitivity Check Index, *HOMA-IR* Homeostasis model assessment of insulin resistance, *Hb A1C* Glycosylated hemoglobin, *FSI* Fasting serum insulin, *FBS* Fasting serum insulin, *MET* metabolic equivalent

*p-value < 0.05, **p-value < 0.01

## Discussion

In this study, the mean body fat percentage and free fat mass were significantly different between the two groups (NWO compared to non-NOW) which was concordant with other studies (https://www.hsph.harvard.edu/gsh-lab/research/inflammation/) [27]. Our study showed that the anthropometric indices including waist and hip circumferences were higher in NWO compared to non-NWO, while waist-to-hip ratio did not differ significantly between the two groups. Also, non-significant differences were observed in the concentrations of total cholesterol, LDL cholesterol, HDL cholesterol, triglycerides, and fasting blood glucose between the two groups. In addition, systolic and diastolic blood pressure did not show significant differences between the two groups. The concentration of hemoglobin A1C in normal weight obese individuals was higher than that in non-obese participants.

Screening based on body fat mass and its distribution in people with a normal BMI or slightly higher than normal BMI is considered an important tool for the prevention of diseases associated with obesity.

Previous studies suggest that chronic inflammation is the main characteristic of obesity and metabolic syndrome. This inflammation is in response to internal factors and does not resemble the pattern of classical inflammation. These studies have shown that obesity and excess body fat mass are related to the activation of serine/threonine kinase and lead to inhibition of insulin receptor signaling through phosphorylation of insulin receptor substrate (IRS-1); this process is the main mechanism of insulin resistance and metabolic disorders (https://www.hsph.harvard.edu/gsh-lab/research/inflammation/).

Studies have shown that obesity and excess body fat mass especially excess visceral fat mass is associated with hyper insulinemia, glucose intolerance, serum elevated triglycerides, and other parameters of the metabolic syndrome [27].

Concordant with our results, the results of studies by Conus in 2004 [25] and De-lorenzo in 2006 and 2007 [8, 10] on American women, and Italian women showed that differences in fat mass and fat free mass were significant between NWO and non-obese participants, whereas the lipid profile, blood glucose and blood pressure was not different significantly in both groups. However, De-lorenzo demonstrated that waist circumference, hip circumference, and waist-to-hip ratio did not differ in NWO compared to non-obese participants. Perhaps, it is the larger sample size in our study compared to the study by De-lorenzo that can justify this finding.

A cohort study by Maderia et al. in 2013 showed that the mean body mass index, waist circumference, hip circumference, LDL-cholesterol, triglycerides, blood pressure and fasting blood sugar were higher in NWO compared to non-NWO individuals [28]; however, some of these findings are in contrast with the findings of the present study.

Moreover, our study showed that the serum insulin concentration and insulin resistance were higher in NWO compared to non-NOW; however, insulin sensitivity was lower in NWO. Studies by Romero-corral [12] and also Madeira FB confirmed the findings of our study but in the study by De Lorenzo [8], fasting serum insulin concentrations and insulin resistance between NWO and non-obese participants were not significantly different.

On the other hand, our study showed a significant correlation between body fat percentage and serum insulin concentrations and insulin sensitivity after adjusting for BMI. However, there was no significant association between body fat percentage and insulin resistance. The study by Madeira FB demonstrated that normal weight obesity increased the risk of metabolic syndrome, insulin resistance, and secretion of insulin and decreased insulin sensitivity. Based on the findings of these studies and those of the present study and considering the fact that insulin resistance and hyper insulinemia are the main causes of metabolic syndrome [29] and the basis for the development of disorders such as type 2 diabetes, it can be endorsed that NWO is a prognostic risk factor of metabolic syndrome and its related chronic diseases in the future.

## ComplementC3 and normal weight obesity

### C3 and metabolic syndrome indicators

Our study showed for the first time that serum complement C3 was higher in NWO than non-obese healthy subjects.

Both insulin resistance and metabolic syndrome are associated with increased inflammatory markers. The marker studied more than other ones in such conditions is CRP (C-reactive protein); however, other markers such as leukocyte count, ESR (erythrocyte sedimentation rate), and serum complement C3 have been evaluated. Among these markers, serum complement C3 is strongly associated with insulin resistance, independent of the components of metabolic syndrome [20].

As mentioned in our study, complement C3 and serum insulin concentrations and insulin resistance were higher in NWO than those in non-NWO subjects. Moreover, in this study, we demonstrated a significant and positive correlation between C3 complement and insulin resistance and serum insulin concentrations and also a significant inverse correlation between C3 and insulin sensitivity, confirming the findings of the previously mentioned studies.

In addition to insulin resistance, other components of metabolic syndrome are also related to complement C3. Studies have shown that serum complement C3 is an early marker of metabolic syndrome in obese people [23, 30].

A prospective cohort study conducted by Onatet alto assessed serum complement C3 as a risk factor for coronary heart disease on 756 men and women. The results showed that complement C3 was correlated with waist circumference, serum triglyceride, and total cholesterol [14].

In line with these studies, the present study showed that complement C3 was correlated with total cholesterol, LDL cholesterol, triglycerides and waist circumference, some of which are components of the metabolic syndrome although no correlation was found between other parameters of metabolic syndrome and serum C3.

### Complement C3, body composition and obesity

Obesity is a disease with mild inflammation that always stimulates the components of the immune system. On the other hand, the complement system is part of the innate immune system and a chronic increased immune response can lead to obesity and metabolic syndrome [18]. Research shows that serum complement C3 has a significant correlation with obesity and also high gene expression complement C3 is observed in abdominal adipose tissue [31]. Our study revealed that serum complement C3 had a significant correlation with body fat percentage in NWO (OR = 1.086 s).

The C3 cleavage product is Acylation Stimulating Protein (ASP) that acts as a metabolic paracrine factor and stimulates insulin secretion. Also, ASP increases fat storage by stimulating triacylglycerol acyl transfer as enzymes in the human adipose tissue. A study showed that ASP deficiency in mice resulted in resistance to weight gain despite receiving a high-fat diet and a high dietary intake [32–35].

A bulk of studies have shown that C3 predicts future cardiovascular events in men and women [36]. These findings as well as other studies suggest a significant relationship between C3 and diabetes in the future [18].

## Conclusion

Since, despite a normal BMI, a high body fat mass leads to increased levels of pro inflammatory cytokines, increased serum insulin concentrations, insulin resistance, and decreased insulin sensitivity, obese women with normal weight may be at risk for metabolic disorders and metabolic syndrome. Finally, it can be asserted that the definition of obesity based on BMI may not reflect the risks associated with it and that individuals with a normal BMI and high body fat percentage can be at risk for metabolic syndrome, cardio metabolic disorders, systemic inflammation and mortality.

Further studies are required to assess the complex interactions between body composition, fat distribution, muscle mass, and metabolism and their impact on the risk factors for chronic diseases.

### Limitation

The measurement of body composition using BIA method is the limitation of our study. Due to the budget constraints, we could not use DEXA that is a gold standard method.

## Abbreviations

BMI: Body mass index
CI: Confidence interval
FM: Fat mass
hsCRP: High sensitivity C reactive protein
IQR: Interquartile range
NWO: Normal weight obese
SD: Standard deviation
